# Acute and subchronic toxicity of 20  kHz and 60  kHz magnetic fields in rats

**DOI:** 10.1002/jat.3161

**Published:** 2015-05-17

**Authors:** Izumi Nishimura, Atsushi Oshima, Kazumoto Shibuya, Takashi Mitani, Tadashi Negishi

**Affiliations:** ^1^ Environmental Science Research LaboratoryCentral Research Institute of Electric Power IndustryChibaJapan; ^2^ Contract Testing DepartmentNippon Institute for Biological ScienceTokyoJapan

**Keywords:** magnetic field, intermediate frequency, non‐ionizing radiation, acute toxicity, subchronic toxicity, rat, risk assessment

## Abstract

Despite increasing use of intermediate frequency (IF) magnetic fields (MFs) in occupational and domestic settings, scientific evidence necessary for health risk assessments of IF MF is insufficient. Male and female Crl:CD(SD) rats (12 per sex per group) were exposed to 20 kHz, 0.20 mT(root mean square, rms) or 60 kHz, 0.10 mT(rms) sinusoidal MFs for 22 h day^−1^ for 14 days (acute) or 13 weeks (subchronic). Experiments were duplicated for each frequency to ensure outcome reproducibility, and examinations were blinded for quality assurance. All rats survived without significant clinical signs until the end of experiments. Some changes in body weight between the MF‐exposed and control groups were observed over the course of exposure, although the directions of the changes were inconsistent and not statistically significant after subchronic exposure. There were significant differences between MF‐exposed and control groups in some organ weights and parameters in hematology and clinical chemistry, but these were minor in magnitude and not repeated in duplicate experiments. Histopathological findings reflecting toxicity were sporadic. Frequencies of other findings were similar to historic data in this rat strain, and findings had no specific relationship to changes in organ weight or parameters of hematology and clinical chemistry in each animal. The changes observed throughout this study were considered biologically isolated and were attributable to chance associations rather than to MF exposure. The results, in particular the histopathological evidence, indicate an absence of toxicity in IF MF‐exposed rats and do not support the hypothesis that IF MF exposure produces significant toxicity. Copyright © 2015. The Authors. *Journal of Applied Toxicology* Published by John Wiley & Sons Ltd.

## Abstract 2

Despite increasing use of intermediate frequency (IF) magnetic fields (MFs) in occupational and domestic settings, scientific evidence is insufficient for IF MF health risk assessments. Rats were exposed to 20 kHz or 60 kHz sinusoidal MFs for 22 h day^−1^ for 14 days (acute) or 13 weeks (subchronic). MF‐exposed rats did not exhibit significant and reproducible changes in body and organ weights, hematology, clinical chemistry and histopathology. Our results indicate that IF MF exposure does not carry a significant health risk to mammals.

## Introduction

The World Health Organization (WHO) conducted a risk assessment of extremely low frequency (<100 kHz) magnetic field (MF) exposure, and subsequently published Environmental Health Criteria (EHC) 238 (WHO, 2007). EHC 238 stated that MFs < 100 kHz have stimulating effects on excitable cells present in tissues such as muscles and nerves. Based on the criteria, the International Commission on Non‐Ionizing Radiation Protection (ICNIRP) set exposure guidelines for MF of < 100 kHz (ICNIRP, 2010), while exposure guidelines ranging from 100 kHz to 300 GHz (radio frequency range) are based on the thermal effects caused by MF exposure (ICNIRP, 1998, 2009). However, EHC 238 assessed health risk based on the biological effects of MFs mostly on power frequencies (50 and 60 Hz) and other frequencies of < 300 Hz. The WHO (2007) and Litvak *et al*. (2002) remarked on the lack of sufficient scientific evidence for health risk assessments of intermediate frequency (IF) MFs from 300 Hz up to 100 kHz.

In 1996, Robertson *et al*. conducted acute and subchronic toxicity studies on very intense and graduated IF MF exposures. They exposed male and female B6C3F1 mice to 0.08, 0.28 or 1.0 mT(root mean square, rms) sinusoidal MFs at 10 kHz for 14 or 90 days, and failed to demonstrate any biological changes related to toxicity produced by IF MF exposure. The modeled 10 kHz sinusoidal MF is employed in inductively coupled power transmission (or wireless/contactless power transfer) systems that are used to transfer electric power efficiently through large gaps between pairs of coils (Boys *et al*., 2000; Budhia *et al*., 2009) in industrial machinery and on some types of electric vehicles and home appliances.

Another group of studies on IF MF toxicity (Kim *et al*., 2006; Lee *et al*., 2006, 2010; Svedenstål and Johanson, 1998a, 1998b) examined MFs associated with video display terminals (VDTs), which emit weak MFs of at most 10 μT(peak‐to‐peak, pp); these are typically about 20 kHz, with complex sawtooth waveforms. VDT MF toxicity studies involved exposure ranging from acute to chronic (20 days to > 2 years), in strains of mice and rats. These studies consistently showed no effects of VDT MF on body and organ weights, hematological and blood chemistry parameters, or histopathological lesions. However, the MFs employed in most VDT MF studies were weak, ranging from 0.00625 to 0.015 mT(pp).

Other than inductively coupled power transmissions and VDTs, instruments such as magnetic resonance imaging machines, induction heaters and industrial welding machines have been reported to be sources of relatively high‐intensity IF MF emissions (Decat *et al*., 2006; Floderus *et al*., 2002; Stuchly and Lecuyer, 1985). One widely spread group of devices utilizing IF MFs is commercial and domestic induction heating (IH) cookers or ovens (ICNIRP, 2008). IH cooking generates vertical spatially varying IF MFs at a fundamental frequency, such as 20 kHz or 60 kHz, and associated harmonic frequencies; these are used together for cooking (Fujita *et al*., 2007; Yamazaki *et al*., 2004). MFs for IH cooking are emitted from the hob and converted to magnetically induced current in the pan, which produces heat for cooking. Field leakage should be weak when the IH apparatus is properly used (FOPH, 2011; Kos *et al*., 2011); however, given that in 2010 the ICNIRP general public exposure guidelines for MF > 3 kHz were revised from 0.00625 to 0.027 mT(rms), rodent toxicity studies of MF exposure above the level are necessary for human health risk assessment.

The aim of this study was to determine whether acute (14 days) or subchronic (13 weeks) exposure to IF MFs produced toxicological effects in the rat. These exposure periods were chosen to be comparable to a previous toxicity study of 10 kHz MF (Robertson *et al*., 1996). We chose 20 and 60 kHz in accordance with our previous reproductive toxicity studies (Nishimura *et al*., 2011, 2012), because these frequencies are typically employed in commercial IH cookers and wireless charging for electric shuttle buses (Tell *et al*., 2014). Sixty kHz is the third harmonic of the 20 kHz frequency and not been examined for acute and subchronic toxicity. Animals were exposed to the maximum MF strength achievable at our facilities at the respective frequencies for hazard identification. Fields of 0.20 mT(rms) at 20 kHz or 0.10 mT(rms) at 60 kHz were generated, which are sufficiently greater than the ICNIRP exposure guidelines (ICNIRP, 2010). A sinusoidal waveform was adopted because of its clear definition and reproducibility. Experiments were performed in duplicate at each frequency to confirm the reproducibility of the outcomes. All examinations after necropsy were conducted in a treatment‐blind manner at an independent GLP‐licensed laboratory for quality assurance.

## Materials and methods

### Experimental design

Because we used two separate IF MF facilities for 20 kHz and 60 kHz exposure that were identical in appearance, but not exchangeable, a maximum exposure dose of only one frequency was applied in a single experiment. When a facility was used for an MF‐exposed group, the other facility was used for the concurrent control (sham‐exposed) group.

This study comprised 10 independent (six acute and four subchronic) experiments and each employed the same experimental protocol. Two of the six acute experiments were conducted as sham–sham studies, where animals were placed in each facility but not exposed to MF, to address concerns regarding the equivalency of the two facilities. For each exposure period (14 days or 13 weeks), two identical experiments (experiments 1 and 2) were conducted using 20 kHz or 60 kHz MF exposure, respectively, to confirm the reproducibility of the outcomes. Irreproducible biological responses to MFs have become an issue in this research field due to inter‐ and intralaboratory variability, and this was of particular concern in our studies due to the limited number of exposure doses in each frequency.

### Animals

The institutional Animal Experiment Committee of the Environmental Science Research Laboratory, Central Research Institute of Electric Power Industry, approved all of the animal experimental procedures used in this study in accordance with the Committee's guidelines under the Japanese Law Concerning the Protection and Management of Animals. Following the Organization for Economic Co‐operation and Development (OECD, 1998) and International Conference on Harmonization (ICH, 2009) guidelines, we exposed both sexes of animals to IF MF for 14 days and 13 weeks for acute and subchronic toxicity studies, respectively.

Five‐week‐old SPF/VAF Crl:CD(SD) rats were purchased from Charles River Laboratories Japan Inc. (Ibaraki, Japan). The rats were quarantined for 1 week in the barrier system at the magnetic field exposure facility, where background MFs were extremely low (< 0.001 μT(rms) at 20 kHz and 60 kHz, and 0.03 μT(rms) at 50 Hz). Room air was HEPA‐filtered, and room temperature and humidity were maintained at 23 ± 2 °C and 50 ± 20%, respectively. The light cycle was 12 h/12 h (on from 07.00 to 19.00 h). The rats were housed in polycarbonate cages (23 cm width × 33 cm depth × 17 cm height; Tokiwa Kagaku Kikai Co. Ltd., Tokyo, Japan) and allowed access to food pellets (CRF‐1, 15 kGy irradiated; Oriental Bioservice, Kyoto, Japan) and water (distilled, autoclaved, 10 ppm chlorine, adjusted to pH 3 by 1 N HCl) ad libitum. Commercial wood chips (White Flake, 30 kGy‐irradiated, Charles River Laboratories Japan Inc.) were used as bedding material. Cage positions within and among the racks were changed twice per week to minimize the influence of subtle environmental differences, such as illumination and airflow. On the last day of the quarantine period, the male and female rats were weighed and randomly assigned to either the MF‐exposed (12 per sex) or control (12 per sex) groups, stratified by body weight.

### Magnetic field exposure facility

The MF exposure facility has been explicitly described in previous papers (Nishimura *et al*., 2011, 2012). A pair of exposure facilities, one for 20 kHz and another for 60 kHz, was built in an area where the background MF at 50 Hz was extremely low. Both facilities were identical with respect to appearance, layout, structure and other room conditions, and were located 37.4 m apart to minimize stray IF MFs during exposures. When the exposure coils were activated to produce maximum MFs, the animals in the control facility received < 0.001 μT(rms) MF at 20 kHz or 60 kHz, and 0.03 μT(rms) at 50 Hz. The geomagnetic field at the location was about 48.3 μT.

Four square coils (Merritt *et al*., 1983) were layered horizontally to produce uniform vertical MFs in a large cubic space (100 × 100 × 100 cm) that was located within the coils. In the designated exposure space, three wooden racks were equipped to accommodate up to 36 rat cages: 12 on each rack in a three‐row × four‐column layout. The electric currents used to produce MFs were maintained within ± 0.2% of the target values during exposure. MFs were calibrated before and after experiments based on 51 spot measurements (17 spots × 3 racks) within the exposure space. The MF variability within the exposure spaces was < 3% in both coil systems at both frequencies (Yamazaki *et al*., 2007). Harmonic components were negligible, at 1 : 100–1 : 1000 of each primary frequency.

The level of ultrasound noise created by the vibration of the coils during MF exposure was measured up to 100 kHz. The recorded background noise level was very low in the animal exposure space. Direct detection of acoustic noise levels was also performed using laser light diffraction. The sound levels for both MF exposures were found to be lower than the background air‐conditioning noise. Coil vibrations were measured using a laser Doppler vibration sensor. The vibrating velocity and acceleration of coils were negligible, and field‐associated heat from the coil bobbins was not appreciable. The room temperature was continuously measured during experiments, and it did not change during the MF exposure.

Rats were exposed to 20 kHz, 0.20 mT(rms) MFs, to 60 kHz, 0.10 mT(rms) MFs, or to the facility with no MF (sham‐exposed) for 22 h day^−1^ (11.00–09.00 h) for 14 days or 13 weeks. The electric current in the exposure coils, which is linearly related to MF production, was monitored continuously and recorded every 10 min by a data logger. Animal care and clinical observations were conducted daily during the 2 h when animals were not exposed to MFs. This exposure regime was maintained throughout the course of the study. Rats were individually housed and specially made polycarbonate cage lids, glass water nozzles and glass pellet containers were used instead of the standard metal versions to avoid induced current during MF exposure.

### Clinical observations

Animals were observed daily for morbidity and clinical signs of toxicity throughout the exposure periods, and abnormal signs were recorded. Body weights were measured at the initiation and termination of exposure periods, on the first, third and seventh days of acute exposure, or once a week during subchronic exposure. Food and water consumption were not measured because the flat cage lids did not accommodate the containers required for food and water consumption measurements. The animals were fasted overnight before necropsy and blood collection.

### Hematological examination

On the termination of exposure, all animals were anesthetized by isoflurane inhalation. Blood samples were drawn from the abdominal aorta and placed into tubes containing the anticoagulant, EDTA‐2K. An automated analyzer (SF‐3000; Sysmex Co., Hyogo, Japan) was used to measure hematological parameters, including erythrocyte count, hemoglobin concentration, hematocrit percentage, mean corpuscular volume (MCV), mean corpuscular hemoglobin (MCH), mean corpuscular hemoglobin concentration, platelet count, reticulocyte count (RET) and leukocyte count. In subchronic toxicity studies, differential leukocyte counts and morphological evaluations of blood cells were performed under the microscope on blood smears stained with Wright–Giemsa.

### Clinical chemistry examination

Aliquots of blood samples for clinical chemistry examination were placed in tubes containing heparin sodium and centrifuged, and the plasma was separated. Clinical chemistry variables were measured using an automated chemistry analyzer (7060; Hitachi Ltd., Tokyo, Japan). Parameters included blood glucose (BGLU), total protein (TP), albumin, albumin/globulin ratio, total cholesterol (TCHO), triglycerides, total bilirubin, creatinine (CRE), blood urea nitrogen, aspartate aminotransferase (AST), alanine aminotransferase (ALT), alkaline phosphatase (ALKP), calcium and inorganic phosphoric acid (IPHS). Plasma sodium, potassium and chloride were measured by an electrolyte analyzer (EA07; Atwill Corp., Kanagawa, Japan).

### Gross pathological examination and organ weight measurement

Complete gross postmortem observations were performed on all animals, and abnormalities were recorded. Absolute organ weights were measured, and organ weights relative to the body weight at necropsy were calculated for the brain, heart, lung with bronchus, thymus, liver, kidneys, adrenal glands, spleen and either the testes or the ovaries and uterus.

### Histopathological examination

The following organs and tissues were obtained from all animals: the heart, aorta, spleen, thymus, bone marrow (sternum and right thigh bone), submandibular lymph node, mesenteric lymph node, trachea, lung with bronchus, salivary gland (mandibular gland and sublingual gland), parotid gland (left), tongue, esophagus, stomach, duodenum, jejunum, ileum, cecum, colon, liver, pancreas, kidneys (left and right), urinary bladder, testes (left and right), epididymides (left and right), seminal vesicle, prostate gland, ovaries (left and right), uterus, vagina, brain, spinal cord, sciatic nerve (right), eyes (left and right), pituitary, thyroid with parathyroid gland, adrenal glands (left and right), skeletal muscle (right thigh), skin (back) and mammary gland. These tissue samples were fixed with 10% neutral buffered formalin solution, embedded in paraffin, sectioned and stained with hematoxylin and eosin for microscopic examination. All gross lesions, as defined by the study pathologist, were also included in the examination. All examinations and measurements after exposure, as well as statistical analyses, were conducted by an independent GLP‐licensed laboratory that was blind to the treatment conditions.

### Statistical analyses

Data were presented as the group means ± SD. Welch's *t*‐test (two‐tailed test) was used for statistical comparison of the MF‐exposed and control group data, with a significance threshold of *P* < 0.05. The data analyzed included body weight, organ and tissue weight, ratio of organ or tissue to body weight, and parameters from hematological and clinical chemistry examinations. Fisher's exact test was applied to evaluate group differences in mortality, clinical findings, gross pathological findings and histopathological findings, with differences between groups considered significant at *P* < 0.05 (one‐tailed test). Statistical analyses were performed using SAS (SAS Institute Inc., Cary, NC, USA) and EXSUS (Arm Co., Ltd., Osaka, Japan) software.

## Results

### Mortality, clinical findings and body weight

In the acute exposure experiments, including the sham–sham experiments, no mortality or abnormal clinical findings were observed in MF‐exposed or control groups of either sex after exposure to 20 kHz or 60 kHz MFs. The body weights of the rats in the MF‐exposed and control groups were similar over the 14 days of experimentation.

In the subchronic exposure experiments, one MF‐exposed female rat in experiment 1 of the 60 kHz MF exposure study exhibited a muzzle nodule after 7 weeks of exposure; and this rat grew normally and continuously gained weight until the termination of exposure. Other than this incidence, no mortality or abnormality was recorded in any animal. The body weights of male and female rats are shown in Table 1. There were small but statistically significant changes in weight in the MF‐exposed male rats in experiment 1 of the 20 kHz and 60 kHz MF exposure studies, which showed a respective decrease and increase in weight, as compared with control rats. These weight differences were observed throughout the exposure period, but were no longer statistically significant after 9 weeks in the group exposed to 20 kHz MF, and after 8 weeks in the group exposed to 60 kHz MF. No other differences in body weight between groups in any experiment were observed.

**Table 1:** Body weights (g) of rats exposed to an intermediate frequency magnetic field for 13 weeks (mean ± SD, *n* = 12)

Exposure week	20 kHz, 0.2 mT				60 kHz, 0.1 mT			
	Experiment 1		Experiment 2		Experiment 1		Experiment 2	
	Control	Exposed	Control	Exposed	Control	Exposed	Control	Exposed
Male								
1	209 ± 5	208 ± 5	205 ± 6	206 ± 6	193 ± 7	194 ± 7	197 ± 5	198 ± 5
2	278 ± 10	275 ± 7	268 ± 11	270 ± 10	255 ± 11	259 ± 10	259 ± 8	261 ± 7
3	341 ± 13	335 ± 11	325 ± 17	327 ± 18	308 ± 17	320 ± 13	313 ± 11	317 ± 16
4	396 ± 19	381 ± 13*	371 ± 24	374 ± 24	351 ± 24	369 ± 17*	359 ± 13	359 ± 23
5	440 ± 22	421 ± 17*	412 ± 32	413 ± 27	389 ± 29	411 ± 20*	396 ± 15	397 ± 30
6	478 ± 27	455 ± 21*	445 ± 38	447 ± 32	421 ± 33	445 ± 22*	430 ± 19	430 ± 35
7	510 ± 29	486 ± 24*	475 ± 42	474 ± 38	448 ± 37	474 ± 24*	457 ± 23	458 ± 37
8	541 ± 33	513 ± 28*	501 ± 47	497 ± 40	473 ± 42	500 ± 24	481 ± 26	479 ± 44
9	567 ± 37	540 ± 30	524 ± 52	519 ± 46	493 ± 45	519 ± 25	503 ± 30	500 ± 49
10	589 ± 39	561 ± 36	541 ± 55	536 ± 46	509 ± 45	539 ± 27	522 ± 31	520 ± 52
11	609 ± 44	582 ± 37	563 ± 57	553 ± 51	525 ± 47	557 ± 28	542 ± 32	536 ± 57
12	627 ± 48	600 ± 41	578 ± 61	569 ± 55	541 ± 51	573 ± 29	556 ± 34	549 ± 63
13	642 ± 50	616 ± 44	593 ± 65	583 ± 59	554 ± 53	585 ± 31	567 ± 37	563 ± 64
Female								
1	167 ± 4	168 ± 4	161 ± 6	159 ± 7	150 ± 6	150 ± 7	159 ± 6	160 ± 5
2	195 ± 9	201 ± 6	188 ± 10	189 ± 9	177 ± 11	176 ± 10	184 ± 6	188 ± 5
3	228 ± 12	233 ± 8	218 ± 12	218 ± 12	203 ± 11	202 ± 13	211 ± 7	212 ± 8
4	253 ± 12	256 ± 10	244 ± 18	239 ± 14	228 ± 19	226 ± 15	234 ± 11	239 ± 11
5	274 ± 16	277 ± 11	260 ± 19	256 ± 17	247 ± 22	244 ± 18	253 ± 14	255 ± 12
6	292 ± 17	293 ± 14	274 ± 22	275 ± 20	261 ± 27	259 ± 22	267 ± 14	271 ± 14
7	307 ± 18	312 ± 16	288 ± 23	288 ± 22	276 ± 27	273 ± 24	280 ± 12	281 ± 17
8	322 ± 17	325 ± 18	298 ± 22	301 ± 27	285 ± 30	283 ± 24	291 ± 14	293 ± 17
9	333 ± 22	335 ± 18	305 ± 25	309 ± 29	295 ± 30	292 ± 27	300 ± 16	302 ± 18
10	342 ± 22	348 ± 19	312 ± 28	317 ± 31	307 ± 33	302 ± 29	309 ± 15	310 ± 20
11	352 ± 22	358 ± 21	322 ± 30	327 ± 33	316 ± 34	311 ± 28	314 ± 16	318 ± 21
12	358 ± 23	366 ± 21	329 ± 31	334 ± 35	322 ± 35	316 ± 33	320 ± 17	327 ± 21
13	365 ± 25	375 ± 23	333 ± 32	339 ± 36	327 ± 36	324 ± 33	327 ± 19	333 ± 21

* Significantly different (*P* < 0.05) from the relevant control group.

### Hematology

In sham–sham experiments, the male rats in the 60 kHz MF facility had MCV and MCH significantly lower than those in the 20 kHz MF facility. However, the differences were relatively small (MCV: 61.9 ± 1.3 vs. 63.3 ± 1.5 fl; MCH: 21.3 ± 0.6 vs. 21.9 ± 0.5 pg) and not reproduced in replicate experiments or in female rats. No other hematological variables showed significant differences between the two sham–sham groups. These results indicated that environmental differences between the exposure facilities did not affect hematology in the rats.

In acute exposure experiments, the 60 kHz MF‐exposed female rats had white blood cells significantly lower than that of the control rats. However, the means showed large SDs and a relatively small difference (90.4 ± 13.2 vs. 111.6 ± 25.4 10^2^ µl^−1^), and this observation was not reproduced in replicate experiments or in male rats. Other hematological parameters assayed did not show significant changes between groups in any acute exposure experiment.

Table 2 shows hematological data from subchronic exposure experiments. There were some increases and decreases in the measured parameters in both sexes of animals in both MF frequency exposure groups. However, the direction of these changes was not consistent among experimental replicates, different sexes of animals and MF frequencies. In addition, no MF‐exposed animals showed multiple abnormal values over a series of hematological variables.

**Table 2:** Hematology in rats exposed to an intermediate frequency magnetic field for 13 weeks (mean ± SD, *n* = 12)

	20 kHz, 0.2 mT				60 kHz, 0.1 mT			
	Experiment 1		Experiment 2		Experiment 1		Experiment 2	
	Control	Exposed	Control	Exposed^a^	Control	Exposed	Control	Exposed
Male								
RBC (10^4^ µl^−1^)	862 ± 28	887 ± 41	853 ± 38	840 ± 46	880 ± 51	868 ± 36	873 ± 27	882 ± 32
HGB (g dl^−1^)	15.4 ± 0.5	16.1 ± 0.6**	15.8 ± 0.6	15.5 ± 0.7	15.6 ± 0.6	15.4 ± 0.5	15.6 ± 0.5	15.7 ± 0.5
HCT (%)	46.4 ± 1.4	48.6 ± 2.0**	45.8 ± 1.9	44.6 ± 2.5	47.7 ± 2.5	46.6 ± 1.9	47.1 ± 1.8	47.5 ± 1.9
MCV (fl)	53.8 ± 1.1	54.8 ± 1.2*	53.8 ± 1.0	53.1 ± 0.9	54.2 ± 1.6	53.7 ± 1.8	53.9 ± 1.4	53.9 ± 1.5
MCH (pg)	17.9 ± 0.5	18.2 ± 0.6	18.5 ± 0.5	18.4 ± 0.5	17.7 ± 0.6	17.8 ± 0.6	17.9 ± 0.4	17.8 ± 0.4
MCHC (g dl^−1^)	33.3 ± 0.4	33.1 ± 0.5	34.4 ± 0.6	34.8 ± 0.7	32.7 ± 0.7	33.1 ± 0.6	33.1 ± 0.7	33.1 ± 0.7
PLT (10^4^ µl^−1^)	105.2 ± 11.8	98.3 ± 13.3	99.8 ± 9.6	106.0 ± 12.2	99.3 ± 17.8	104.4 ± 10.1	105.8 ± 18.9	104.5 ± 13.4
RET (‰)	13 ± 3	12 ± 2	25 ± 4	27 ± 6	14 ± 2	13 ± 2	17 ± 4	16 ± 5
WBC (10^2^ µl^−1^)	100.3 ± 21.2	106.8 ± 26.1	79.7 ± 27.1	80.5 ± 21.0	89.0 ± 20.3	88.4 ± 18.9	91.8 ± 19.7	78.9 ± 26.0
Differential leukocyte counts (%)								
Neutrophils, band	0.4 ± 0.4	0.5 ± 0.2	0.7 ± 0.7	1.6 ± 1.4	0.5 ± 0.3	0.5 ± 0.4	1.3 ± 0.8	1.1 ± 0.5
Neutrophils, segmented	7.9 ± 3.0	9.8 ± 3.7	9.0 ± 4.6	9.1 ± 3.7	9.5 ± 3.2	9.8 ± 4.4	10.5 ± 3.7	9.1 ± 3.8
Lymphocytes	87.3 ± 3.9	86.7 ± 4.0	87.0 ± 5.0	86.0 ± 5.5	86.3 ± 3.7	86.3 ± 5.4	84.0 ± 4.9	85.6 ± 3.2
Monocytes	2.2 ± 0.8	1.7 ± 0.8	0.6 ± 0.7	0.8 ± 0.9	1.8 ± 0.9	1.8 ± 1.0	2.0 ± 1.0	2.5 ± 0.7
Eosinophils	2.1 ± 1.1	1.2 ± 0.9*	2.7 ± 1.2	2.5 ± 1.7	1.9 ± 1.2	1.7 ± 1.3	2.0 ± 1.3	1.7 ± 0.9
Basophils	0.1 ± 0.2	0.0 ± 0.1	0.0 ± 0.0	0.0 ± 0.0	0.0 ± 0.0	0.0 ± 0.1	0.1 ± 0.2	0.0 ± 0.1
Others	0.0 ± 0.0	0.0 ± 0.0	0.0 ± 0.0	0.0 ± 0.0	0.0 ± 0.0	0.0 ± 0.0	0.0 ± 0.0	0.0 ± 0.0
Female								
RBC (10^4^ µl^−1^)	778 ± 35	772 ± 30	773 ± 34	764 ± 43	742 ± 40	774 ± 26*	786 ± 19	775 ± 35
HGB (g dl^−1^)	15.5 ± 0.4	15.2 ± 0.5	15.0 ± 0.3	14.6 ± 0.5*	14.6 ± 0.5	15.1 ± 0.4*	15.4 ± 0.5	15.3 ± 0.5
HCT (%)	44.5 ± 1.6	44.1 ± 1.5	42.3 ± 1.5	41.8 ± 1.9	41.1 ± 1.8	42.9 ± 1.5*	44.1 ± 1.9	43.9 ± 1.8
MCV (fl)	57.2 ± 1.5	57.1 ± 1.5	54.8 ± 0.9	54.8 ± 1.6	55.4 ± 1.6	55.4 ± 1.3	56.1 ± 1.4	56.7 ± 1.6
MCH (pg)	19.9 ± 0.7	19.7 ± 0.5	19.4 ± 0.7	19.2 ± 0.9	19.7 ± 0.7	19.5 ± 0.7	19.6 ± 0.5	19.8 ± 0.8
MCHC (g dl^−1^)	34.8 ± 0.9	34.5 ± 0.6	35.5 ± 0.9	34.9 ± 0.9	35.5 ± 0.7	35.2 ± 0.7	35.0 ± 0.8	34.9 ± 0.9
PLT (10^4^ µl^−1^)	100.6 ± 13.8	97.8 ± 26.1	103.2 ± 10.7	98.0 ± 9.4	103.0 ± 8.0	103.7 ± 7.7	94.5 ± 15.6	97.8 ± 9.5
RET (‰)	14 ± 3	13 ± 2	32 ± 6	30 ± 4	12 ± 1	13 ± 2	19 ± 5	22 ± 4
WBC (10^2^ µl^−1^)	54.7 ± 15.3	53.7 ± 16.2	34.8 ± 13.3	36.8 ± 11.9	50.0 ± 12.4	51.9 ± 14.9	55.2 ± 11.5	61.2 ± 14.0
Differential leukocyte counts (%)								
Neutrophils, band	0.5 ± 0.4	0.6 ± 0.4	1.4 ± 0.9	1.3 ± 1.1	0.6 ± 0.3	0.9 ± 0.6	1.4 ± 0.9	1.4 ± 0.7
Neutrophils, segmented	7.8 ± 3.2	8.7 ± 3.0	11.0 ± 6.0	10.3 ± 5.4	12.0 ± 5.9	12.1 ± 5.1	7.5 ± 2.0	6.8 ± 2.8
Lymphocytes	87.2 ± 3.9	85.6 ± 5.0	84.2 ± 6.3	86.0 ± 5.2	84.1 ± 5.7	83.7 ± 5.6	87.2 ± 2.9	88.4 ± 3.5
Monocytes	1.3 ± 0.9	2.5 ± 0.8**	0.9 ± 0.7	0.4 ± 0.4*	1.6 ± 0.7	0.9 ± 0.3**	1.2 ± 0.6	0.9 ± 0.4
Eosinophils	3.3 ± 1.8	2.5 ± 1.6	2.5 ± 1.4	2.0 ± 1.7	1.6 ± 0.9	2.4 ± 1.1	2.6 ± 1.2	2.5 ± 1.1
Basophils	0.0 ± 0.1	0.1 ± 0.2	0.0 ± 0.0	0.0 ± 0.0	0.1 ± 0.2	0.0 ± 0.0	0.1 ± 0.2	0.0 ± 0.1
Others	0.0 ± 0.0	0.0 ± 0.0	0.0 ± 0.0	0.0 ± 0.0	0.0 ± 0.0	0.0 ± 0.0	0.0 ± 0.0	0.0 ± 0.0

HCT, hematocrit percentage; HGB, hemoglobin concentration; MCH, mean corpuscular hemoglobin; MCHC, mean corpuscular hemoglobin concentration; MCV, mean corpuscular volume; PLT, platelet count; RBC, erythrocyte count; RET, reticulocyte count; WBC, leukocyte count.

a One male rat's sample in this group was not examined due to clotting.

* Significantly different (*P* < 0.05) from the relevant control group.

** Significantly different (P < 0.01) from the relevant control group.

### Clinical chemistry

In sham–sham experiments, there were some statistically significant group differences in the measured parameters. BGLU, TP, TCHO and IPHS were increased in the 60 kHz MF facility group as compared with the 20 kHz MF facility group, and CRE, ALT and plasma sodium were decreased. Some of these differences were relatively small (data not shown), most were not common to both sexes, and none were reproduced in replicate experiments. These isolated changes were observed in 17 parameters, but no other significant group differences were observed for clinical chemistry parameters. Overall results did not indicate that rats bred in the two facilities differed in clinical chemistry.

In acute exposure experiments, TP (5.32 ± 0.20 vs. 5.12 ± 0.23 g dl^−1^) and TCHO (63.4 ± 13.9 vs. 50.8 ± 5.9 mg dl^−1^) levels were increased in 20 kHz MF‐exposed male rats in comparison with control rats, and ALKP (590 ± 97 vs. 691 ± 130 IU l^−1^) was decreased. In addition, AST (62.3 ± 3.6 vs. 68.8 ± 9.7 IU l^−1^) was decreased in male rats exposed to 60 kHz MF. However, these changes in clinical chemistry parameters were not reproduced in replicate experiments or in female rats. No other significant differences between the study groups were observed.

Clinical chemistry data from subchronic exposure experiments are shown in Table 3. There were some increases and decreases in the measured parameters in both sexes of animals in both MF frequency exposure groups. Again, these changes were inconsistent among experimental replicates, sexes of animals and MF frequencies. Group differences in inorganic ions were negligible. No MF‐exposed animal showed multiple abnormal values over the range of clinical chemistry parameters.

**Table 3:** Blood biochemistry in rats exposed to an intermediate frequency magnetic field for 13 weeks (mean ± SD, *n* = 12)

	20 kHz, 0.2 mT				60 kHz, 0.1 mT			
	Experiment 1		Experiment 2		Experiment 1		Experiment 2	
	Control	Exposed	Control	Exposed	Control	Exposed	Control	Exposed
Male								
BGLU (mg dl^−1^)	166 ± 18	163 ± 17	187 ± 43	189 ± 39	157 ± 20	157 ± 18	133 ± 20	143 ± 26
TP (g dl^−1^)	6.36 ± 0.30	6.30 ± 0.21	6.10 ± 0.32	6.04 ± 0.28	6.27 ± 0.24	6.46 ± 0.25	6.40 ± 0.40	6.41 ± 0.26
ALB (g dl^−1^)	4.79 ± 0.22	4.81 ± 0.14	4.67 ± 0.24	4.67 ± 0.28	4.87 ± 0.23	4.91 ± 0.17	4.88 ± 0.30	4.86 ± 0.22
A/G ratio	3.07 ± 0.35	3.29 ± 0.46	3.34 ± 0.63	3.44 ± 0.52	3.53 ± 0.40	3.21 ± 0.47	3.25 ± 0.42	3.18 ± 0.39
TCHO (mg dl^−1^)	68.2 ± 18.4	63.0 ± 11.5	59.2 ± 14.4	66.6 ± 15.4	59.1 ± 9.0	64.8 ± 14.3	67.1 ± 16.2	62.5 ± 11.6
TGL (mg dl^−1^)	74.6 ± 22.8	94.5 ± 44.3	78.7 ± 28.5	75.7 ± 41.2	58.5 ± 35.9	60.8 ± 20.8	86.2 ± 39.2	79.9 ± 24.3
TB (mg dl^−1^)	0.086 ± 0.020	0.082 ± 0.014	0.088 ± 0.014	0.080 ± 0.015	0.072 ± 0.016	0.072 ± 0.018	0.079 ± 0.010	0.076 ± 0.019
CRE (mg dl^−1^)	0.193 ± 0.029	0.181 ± 0.035	0.299 ± 0.062	0.287 ± 0.071	0.262 ± 0.028	0.259 ± 0.024	0.242 ± 0.036	0.250 ± 0.030
BUN (mg dl^−1^)	14.4 ± 1.3	13.8 ± 0.9	14.6 ± 1.0	14.2 ± 2.0	14.7 ± 1.6	14.7 ± 1.5	14.6 ± 2.1	14.7 ± 1.1
AST (IU l^−1^)	82.1 ± 21.8	72.5 ± 8.4	74.9 ± 16.0	74.8 ± 12.9	85.8 ± 15.4	94.4 ± 17.7	80.2 ± 17.2	77.0 ± 9.3
ALT (IU l^−1^)	30.2 ± 9.1	31.6 ± 7.5	32.3 ± 6.6	32.4 ± 7.8	28.6 ± 6.3	32.8 ± 5.9	30.0 ± 4.5	31.3 ± 4.5
ALKP (IU l^−1^)	284 ± 54	291 ± 76	286 ± 56	318 ± 64	273 ± 55	280 ± 42	259 ± 43	277 ± 47
CALC (mg dl^−1^)	10.22 ± 0.44	10.20 ± 0.20	10.33 ± 0.27	10.35 ± 0.21	10.51 ± 0.42	10.51 ± 0.30	10.33 ± 0.35	10.36 ± 0.34
IPHS (mg dl^−1^)	5.93 ± 0.58	6.06 ± 0.57	5.77 ± 1.10	5.51 ± 0.59	5.97 ± 0.29	5.82 ± 0.59	5.50 ± 0.60	5.46 ± 0.49
Na (mEq l^−1^)	133 ± 3	135 ± 2	144 ± 1	145 ± 1**	142 ± 1	142 ± 1	144 ± 1	144 ± 1
K (mEq l^−1^)	5.19 ± 0.67	5.36 ± 0.78	4.43 ± 0.28	4.41 ± 0.23	4.02 ± 0.31	4.12 ± 0.22	3.79 ± 0.37	3.72 ± 0.40
Cl (mEq l^−1^)	96 ± 3	98 ± 3	101 ± 1	102 ± 2	104 ± 1	104 ± 1	106 ± 3	105 ± 2
Female								
BGLU (mg dl^−1^)	153 ± 10	161 ± 15	165 ± 19	176 ± 24	138 ± 17	140 ± 19	133 ± 15	135 ± 17
TP (g dl^−1^)	6.85 ± 0.49	7.01 ± 0.50	6.95 ± 0.44	6.85 ± 0.41	7.00 ± 0.40	7.09 ± 0.40	7.24 ± 0.44	7.22 ± 0.33
ALB (g dl^−1^)	5.44 ± 0.38	5.64 ± 0.49	5.79 ± 0.47	5.71 ± 0.39	5.91 ± 0.38	5.91 ± 0.38	5.98 ± 0.45	5.96 ± 0.32
A/G ratio	3.92 ± 0.38	4.26 ± 1.04	5.06 ± 0.91	5.18 ± 0.98	5.51 ± 0.79	5.15 ± 1.12	4.85 ± 0.94	4.81 ± 0.63
TCHO (mg dl^−1^)	80.0 ± 13.9	86.7 ± 29.5	89.5 ± 16.9	93.5 ± 19.9	84.0 ± 15.6	84.7 ± 15.8	86.3 ± 23.6	82.8 ± 13.0
TGL (mg dl^−1^)	61.9 ± 28.9	76.8 ± 46.9	66.0 ± 43.7	63.4 ± 46.6	45.5 ± 26.1	56.0 ± 45.2	61.7 ± 46.9	70.0 ± 28.1
TB (mg dl^−1^)	0.098 ± 0.014	0.109 ± 0.021	0.107 ± 0.014	0.094 ± 0.017	0.100 ± 0.021	0.102 ± 0.022	0.110 ± 0.029	0.108 ± 0.019
CRE (mg dl^−1^)	0.241 ± 0.056	0.230 ± 0.041	0.350 ± 0.030	0.356 ± 0.031	0.328 ± 0.047	0.355 ± 0.044	0.283 ± 0.040	0.323 ± 0.039*
BUN (mg dl^−1^)	13.7 ± 2.9	14.0 ± 2.7	15.0 ± 0.9	14.6 ± 1.3	14.0 ± 1.5	14.7 ± 1.1	13.5 ± 1.6	15.0 ± 1.2*
AST (IU l^−1^)	58.3 ± 7.9	63.7 ± 17.4	65.6 ± 22.1	71.4 ± 30.5	70.8 ± 13.1	76.2 ± 14.8	63.2 ± 7.6	67.7 ± 12.1
ALT (IU l^−1^)	22.2 ± 2.9	28.3 ± 13.6	25.8 ± 8.8	28.0 ± 14.5	23.7 ± 5.3	26.4 ± 7.0	30.4 ± 11.7	24.7 ± 7.5
ALKP (IU l^−1^)	121 ± 31	121 ± 27	117 ± 25	116 ± 21	102 ± 24	110 ± 31	96 ± 15	94 ± 17
CALC (mg dl^−1^)	10.29 ± 0.29	10.38 ± 0.36	10.57 ± 0.28	10.46 ± 0.27	10.69 ± 0.29	10.71 ± 0.26	10.68 ± 0.24	10.77 ± 0.27
IPHS (mg dl^−1^)	4.01 ± 0.70	4.42 ± 1.12	3.53 ± 0.73	3.46 ± 0.69	3.69 ± 0.61	3.94 ± 0.85	4.08 ± 0.90	4.36 ± 0.80
Na (mEq l^−1^)	137 ± 3	138 ± 3	142 ± 1	143 ± 1*	142 ± 1	141 ± 1**	143 ± 1	144 ± 1
K (mEq l^−1^)	5.48 ± 0.56	5.48 ± 0.61	3.57 ± 0.22	3.69 ± 0.14	3.73 ± 0.27	3.91 ± 0.50	4.03 ± 0.21	4.09 ± 0.31
Cl (mEq l^−1^)	104 ± 3	104 ± 2	101 ± 2	103 ± 1*	106 ± 2	106 ± 1	105 ± 2	104 ± 2

A/G ratio, albumin/globulin ratio; ALB, albumin; ALKP, alkaline phosphatase; ALT, alanine aminotransferase; AST, aspartate aminotransferase; BGLU, blood glucose; BUN, blood urea nitrogen; CALC, calcium; Cl, chloride; CRE, creatinine; IPHS, inorganic phosphoric acid; K, potassium; Na, sodium; TB, total bilirubin; TCHO, total cholesterol; TGL, triglycerides; TP, total protein.

* Significantly different (*P* < 0.05) from the relevant control group.

** Significantly different (*P* < 0.01) from the relevant control group.

In the 20 kHz MF experiment 2, RET values in both control and exposed groups of male and female rats were relatively higher than observed in other experiments. The reason for this difference was unclear and it may reflect animal batch variation. Although the animals were younger (9–11 weeks) than those included in the present study, Nagase *et al*. (2000) reported RET of 34‰ in male and 27‰ in female rats of the same strain. We consider that the obtained RET was around the normal range for this strain of rat.

### Gross pathological findings

In sham–sham experiments, a kidney cyst was observed in a male rat held in the 20 kHz MF facility. In the animals in the 60 kHz MF facility, kidney hypoplasia and dilatation of the uterus were observed in a female rat, and brown foci were found in the liver of a different female rat.

In 20 kHz MF acute exposure experiments, dilatation of the renal pelvis and a uterine cyst were found in a female MF‐exposed rat, and a control female had a uterine cyst. In 60 kHz MF exposure experiments, nodules in the spleen and two kidney cysts were recorded in three male rats in the MF‐exposed groups. One control male rat had liver congestion and another had a kidney cyst. One control male rat exhibited a localized loss of fur. Overall, the frequencies of gross pathological findings in sham–sham and acute exposure experiments were low across animals in the control and MF‐exposed groups.

Pathological changes in the subchronic exposure experiments were also very low in frequency, and the changes showed no association with the MF exposure, specific organs or the sex of the animal. These changes were brown foci in the brain, a unilateral kidney cyst, caudate lobe torsion and abnormal lobulation in the liver, muzzle nodules and a pituitary cyst in the 60 kHz MF‐exposed rats. No change was observed in the 20 kHz MF‐exposed rats. Control rats exhibited diverticulum in the ileum and swelling of the medial iliac lymph node. Observations of brown foci in the brain, nodules in the muzzle and swelling of the medial iliac lymph node were toxicologically noteworthy but no statistically significant group differences in the frequencies of any of the findings were evident.

### Organ weight

In sham–sham experiments, there were two statistically significant differences in relative organ to body weights between the study groups. Male rats in the 60 kHz MF facility had a lower relative lung weight than male rats in the 20 kHz MF facility (0.376 ± 0.023 vs. 0.399 ± 0.031%), and female rats in the 60 kHz MF facility had a higher relative spleen weight than female rats in the 20 kHz MF facility (0.226 ± 0.028 vs. 0.204 ± 0.016%). No other significant differences were observed. These results indicated that the breeding environments of the exposure facilities did not influence relative organ weights.

In the acute exposure experiments, four statistically significant differences were found. The relative heart and adrenal gland weights in male rats in the 20 kHz MF‐exposed group were higher than in the control group (heart: 0.369 ± 0.023 vs. 0.349 ± 0.017%; adrenal glands: 0.0188 ± 0.0019 vs. 0.0171 ± 0.0017%). The relative weights of the epididymides and ovaries in rats exposed to 20 kHz MF were lower than the control rats (epididymides: 0.224 ± 0.011 vs. 0.239 ± 0.018%; ovaries: 0.0427 ± 0.0052 vs. 0.0472 ± 0.0052%). No other significant differences were observed.

Table 4 shows the relative organ weights in the subchronic exposure experiments. There were some increases and decreases in both sexes exposed to both frequencies of MF exposure. However, the magnitude of the changes was relatively small, they were inconsistent between experimental replicates, and did not clearly associate with the sex of the animal or the MF frequency. No specific organ weight was affected by exposure to either MF frequency.

**Table 4:** Organ to body weight ratio (%) in rats exposed to an intermediate frequency magnetic field for 13 weeks (mean ± SD, *n* = 12)

	20 kHz, 0.2 mT				60 kHz, 0.1 mT			
	Experiment 1		Experiment 2		Experiment 1		Experiment 2	
	Control	Exposed	Control	Exposed	Control	Exposed	Control	Exposed
Male								
Brain	0.347 ± 0.032	0.364 ± 0.030	0.382 ± 0.040	0.368 ± 0.038	0.400 ± 0.026	0.379 ± 0.026	0.389 ± 0.033	0.394 ± 0.040
Heart	0.280 ± 0.016	0.286 ± 0.017	0.277 ± 0.016	0.277 ± 0.024	0.298 ± 0.023	0.280 ± 0.017*	0.279 ± 0.026	0.282 ± 0.018
Lung, with bronchus	0.241 ± 0.021	0.265 ± 0.016**	0.264 ± 0.032	0.258 ± 0.026	0.288 ± 0.019	0.269 ± 0.030	0.261 ± 0.024	0.260 ± 0.018
Thymus	0.055 ± 0.014	0.060 ± 0.012	0.051 ± 0.013	0.053 ± 0.008	0.061 ± 0.009	0.061 ± 0.017	0.062 ± 0.010	0.054 ± 0.010
Liver	2.56 ± 0.23	2.61 ± 0.19	2.40 ± 0.10	2.39 ± 0.12	2.53 ± 0.17	2.59 ± 0.10	2.57 ± 0.25	2.48 ± 0.22
Kidney, left	0.275 ± 0.022	0.297 ± 0.024*	0.290 ± 0.019	0.294 ± 0.024	0.302 ± 0.022	0.302 ± 0.025	0.298 ± 0.036	0.295 ± 0.019
Kidney, right	0.271 ± 0.020	0.299 ± 0.022**	0.290 ± 0.018	0.293 ± 0.028	0.303 ± 0.024	0.299 ± 0.030	0.294 ± 0.033	0.287 ± 0.021
Adrenal, left	0.0045 ± 0.0004	0.0054 ± 0.0006**	0.0052 ± 0.0008	0.0045 ± 0.0005*	0.0057 ± 0.0010	0.0052 ± 0.0007	0.0051 ± 0.0005	0.0052 ± 0.0008
Adrenal, right	0.0043 ± 0.0004	0.0052 ± 0.0007**	0.0049 ± 0.0007	0.0043 ± 0.0005*	0.0053 ± 0.0011	0.0050 ± 0.0007	0.0048 ± 0.0005	0.0047 ± 0.0007
Spleen	0.152 ± 0.033	0.140 ± 0.020	0.135 ± 0.019	0.131 ± 0.014	0.141 ± 0.011	0.140 ± 0.019	0.132 ± 0.020	0.134 ± 0.013
Testis, left	0.296 ± 0.032	0.300 ± 0.023	0.315 ± 0.036	0.306 ± 0.029	0.333 ± 0.031	0.306 ± 0.024*	0.306 ± 0.033	0.302 ± 0.031
Testis, right	0.296 ± 0.029	0.298 ± 0.021	0.318 ± 0.037	0.307 ± 0.029	0.327 ± 0.031	0.310 ± 0.023	0.306 ± 0.035	0.302 ± 0.034
Female								
Brain	0.577 ± 0.049	0.554 ± 0.031	0.628 ± 0.067	0.611 ± 0.063	0.610 ± 0.067	0.630 ± 0.062	0.642 ± 0.127	0.614 ± 0.054
Heart	0.314 ± 0.022	0.309 ± 0.019	0.313 ± 0.023	0.305 ± 0.022	0.316 ± 0.020	0.318 ± 0.024	0.304 ± 0.021	0.302 ± 0.041
Lung, with bronchus	0.340 ± 0.027	0.341 ± 0.026	0.342 ± 0.036	0.350 ± 0.032	0.348 ± 0.029	0.343 ± 0.028	0.331 ± 0.025	0.323 ± 0.028
Thymus	0.099 ± 0.019	0.091 ± 0.017	0.081 ± 0.010	0.094 ± 0.016*	0.104 ± 0.024	0.094 ± 0.019	0.088 ± 0.020	0.082 ± 0.016
Liver	2.33 ± 0.11	2.43 ± 0.22	2.24 ± 0.12	2.28 ± 0.10	2.43 ± 0.18	2.31 ± 0.18	2.29 ± 0.13	2.23 ± 0.13
Kidney, left	0.291 ± 0.020	0.296 ± 0.023	0.295 ± 0.021	0.302 ± 0.032	0.304 ± 0.034	0.275 ± 0.018*	0.298 ± 0.019	0.287 ± 0.033
Kidney, right	0.301 ± 0.020	0.301 ± 0.024	0.299 ± 0.016	0.310 ± 0.031	0.307 ± 0.032	0.284 ± 0.021	0.307 ± 0.020	0.287 ± 0.035
Adrenal, left	0.0101 ± 0.0011	0.0107 ± 0.0020	0.0096 ± 0.0014	0.0098 ± 0.0016	0.0113 ± 0.0018	0.0109 ± 0.0018	0.0103 ± 0.0014	0.0096 ± 0.0018
Adrenal, right	0.0093 ± 0.0012	0.0099 ± 0.0015	0.0090 ± 0.0014	0.0098 ± 0.0016	0.0110 ± 0.0021	0.0104 ± 0.0019	0.0097 ± 0.0013	0.0094 ± 0.0014
Spleen	0.163 ± 0.023	0.147 ± 0.024	0.157 ± 0.031	0.147 ± 0.015	0.156 ± 0.021	0.154 ± 0.026	0.156 ± 0.019	0.150 ± 0.021
Ovary, left	0.0145 ± 0.0023	0.0129 ± 0.0028	0.0139 ± 0.0029	0.0147 ± 0.0046	0.0122 ± 0.0022	0.0129 ± 0.0019	0.0113 ± 0.0021	0.0122 ± 0.0021
Ovary, right	0.0129 ± 0.0017	0.0130 ± 0.0030	0.0130 ± 0.0028	0.0132 ± 0.0025	0.0124 ± 0.0026	0.0127 ± 0.0024	0.0113 ± 0.0023	0.0120 ± 0.0024
Uterus	0.187 ± 0.066	0.157 ± 0.029	0.203 ± 0.063	0.225 ± 0.061	0.213 ± 0.079	0.220 ± 0.072	0.197 ± 0.047	0.164 ± 0.032

* Significantly different (*P* < 0.05) from the relevant control group.

** Significantly different (*P* < 0.01) from the relevant control group.

### Histopathology

The histopathological findings are shown in Table 5 (male) and Table 6 (female). Although there were some animal batch‐related variations in the frequencies of these findings, none showed statistically significant group differences within an experiment. No specific organ showed accumulated histopathological changes. Brown foci in the brain, nodules in the muzzle and swelling of the medial iliac lymph node observed in gross pathological examinations, were confirmed histopathologically by the observation of astrocytes, squamous cell papilloma and increased lymphocytes, respectively.

**Table 5:** Histopathology in male rats exposed to an intermediate frequency magnetic field for 13 weeks (*n* = 12)

Tissues	Findings	20 kHz, 0.2 mT				60 kHz, 0.1 mT			
		Experiment 1		Experiment 2		Experiment 1		Experiment 2	
		Control	Exposed	Control	Exposed	Control	Exposed	Control	Exposed
Adrenal gland	Fatty change, cortex	1 (8.3)	0	1 (8.3)	2 (16.7)	3 (25.0)	3 (25.0)	3 (25.0)	2 (16.7)
Brain	Astrocytoma	0	0	0	0	0	0	0	1 (8.3)
Eye	Dysplasia, retina	0	0	0	1 (8.3)	0	0	1 (8.3)	0
Heart	Brown pigment deposit	1 (8.3)	0	0	0	0	0	0	0
	Cellular infiltration	0	0	4 (33.3)	5 (41.7)	0	0	0	0
	Cellular infiltration, interstitium	6 (50.0)	5 (41.7)	0	0	3 (25.0)	2 (16.7)	5 (41.7)	4 (33.3)
	Degeneration, myocardium	2 (16.7)	4 (33.3)	2 (16.7)	2 (16.7)	2 (16.7)	2 (16.7)	2 (16.7)	2 (16.7)
	Fibrosis, myocardium	2 (16.7)	5 (41.7)	1 (8.3)	3 (25.0)	0	2 (16.7)	3 (25.0)	2 (16.7)
	Hemorrhage	1 (8.3)	0	0	0	0	0	0	0
Kidney	Basophilic tubular epithelium	6 (50.0)	5 (41.7)	4 (33.3)	5 (41.7)	5 (41.7)	7 (58.3)	7 (58.3)	4 (33.3)
	Calcification	3 (25.0)	1 (8.3)	0	0	0	2 (16.7)	0	0
	Cellular infiltration, interstitium	4 (33.3)	1 (8.3)	0	0	0	0	0	0
	Cyst	0	0	0	0	0	0	0	1 (8.3)
	Dilatation, tubule	0	0	0	0	0	1 (8.3)	0	0
	Eosinophilic body, proximal tubule	0	0	1 (8.3)	0	0	0	8 (66.7)	6 (50.0)
	Protein casts	1 (8.3)	0	0	0	3 (25.0)	1 (8.3)	0	0
Liver	Congestion, caudate lobe (moderate)	0	0	0	0	0	0	0	1 (8.3)
	Fatty change, centrilobule	0	0	0	0	0	0	1 (8.3)	1 (8.3)
	Fatty change, periphery	2 (16.7)	1 (8.3)	0	0	0	0	0	0
	Microgranuloma	1 (8.3)	0	1 (8.3)	1 (8.3)	1 (8.3)	1 (8.3)	0	0
	Necrosis, caudate lobe (severe)	0	0	0	0	0	0	0	1 (8.3)
Lung with bronchus	Aggregation, foam cell	3 (25.0)	5 (41.7)	4 (33.3)	5 (41.7)	5 (41.7)	5 (41.7)	5 (41.7)	5 (41.7)
	Calcification, blood vessel	6 (50.0)	4 (33.3)	2 (16.7)	4 (33.3)	2 (16.7)	2 (16.7)	0	3 (25.0)
	Cellular infiltration	0	1 (8.3)	0	0	1 (8.3)	0	0	0
	Osseous metaplasia, intra‐alveolar	0	0	0	1 (8.3)	1 (8.3)	0	1 (8.3)	2 (16.7)
Mandibular gland	Ectopia, parotid gland	1 (8.3)	1 (8.3)	0	0	1 (8.3)	1 (8.3)	0	1 (8.3)
	Ectopia, sublingual gland	0	0	0	0	1 (8.3)	1 (8.3)	0	0
Pancreas	Basophilic cell focus, acinar cell	0	1 (8.3)	0	0	0	0	0	0
	Cellular infiltration	4 (33.3)	1 (8.3)	1 (8.3)	0	0	1 (8.3)	0	0
	Cellular infiltration (moderate)	1 (8.3)	0	0	0	0	0	0	0
	Fibrosis, islet cell	4 (33.3)	2 (16.7)	0	0	1 (8.3)	2 (16.7)	0	0
	Hemorrhage	2 (16.7)	0	0	0	0	0	0	0
	Hyperplasia, islet cell	0	1 (8.3)	0	0	0	1 (8.3)	0	0
	Inflammation	0	0	0	0	0	0	2 (16.7)	1 (8.3)
Pituitary	Rathke's pouch remnants	1 (8.3)	0	1 (8.3)	1 (8.3)	0	0	1 (8.3)	0
Prostate gland	Aggregation, lymphoid cell	0	0	6 (50.0)	4 (33.3)	4 (33.3)	2 (16.7)	5 (41.7)	4 (33.3)
	Cellular infiltration	3 (25.0)	4 (33.3)	0	0	0	0	0	0
	Inflammation	0	0	0	0	0	0	1 (8.3)	0
Spleen	Brown pigment deposit	0	3 (25.0)	0	0	0	0	0	0
	Extramedullary hematopoiesis	0	0	0	0	2 (16.7)	0	0	0
Sublingual gland	Ectopia, parotid gland	0	0	0	0	1 (8.3)	0	0	0
Testis	Degeneration, seminiferous tubule	1 (8.3)	0	0	0	0	0	0	0
Thyroid	Aggregation, lymphoid cell	0	0	0	0	0	1 (8.3)	0	0
	Ultimobranchial cyst	2 (16.7)	0	1 (8.3)	0	0	0	0	2 (16.7)

Values indicate incidence (%).

Findings are displayed for tissues with at least one incidence of abnormality from a total of 35 tissues examined in each animal.

The following tissues showed no abnormalities and were therefore not included in this table: bone, bone marrow, cecum, colon, duodenum, esophagus, ileum, jejunum, mammary gland, mandibular lymph node, mesenteric lymph node, parathyroid, seminal vesicle, skin/subcutis, spinal cord, stomach, thymus, tongue, trachea and urinary bladder.

**Table 6:** Histopathology in female rats exposed to an intermediate frequency magnetic field for 13 weeks (*n* = 12)

Tissues	Findings	20 kHz, 0.2 mT				60 kHz, 0.1 mT			
		Experiment 1		Experiment 2		Experiment 1		Experiment 2	
		Control	Exposed	Control	Exposed	Control	Exposed	Control	Exposed
Adrenal gland	Fatty change, cortex	0	0	0	1 (8.3)	0	0	0	0
Eye	Dysplasia, retina	0	0	0	0	0	0	1 (8.3)	0
Heart	Cellular infiltration	0	0	1 (8.3)	0	0	0	0	0
	Cellular infiltration, interstitium	3 (25.0)	0	0	0	1 (8.3)	0	0	1 (8.3)
	Degeneration, myocardium	0	0	0	0	0	0	0	1 (8.3)
Ileum	Calcification, Peyer's patch	1 (8.3)	0	0	0	0	0	0	0
	Diverticulum	1 (8.3)	0	0	0	0	0	0	0
Kidney	Basophilic tubular epithelium	2 (16.7)	2 (16.7)	1 (8.3)	1 (8.3)	2 (16.7)	2 (16.7)	1 (8.3)	1 (8.3)
	Calcification	2 (16.7)	3 (25.0)	1 (8.3)	1 (8.3)	1 (8.3)	0	2 (16.7)	0
	Cellular infiltration, interstitium	4 (33.3)	2 (16.7)	0	0	1 (8.3)	1 (8.3)	0	0
	Cellular infiltration, renal pelvis	0	0	0	0	0	0	3 (25.0)	1 (8.3)
	Degeneration, tubular epithelium	0	1 (8.3)	0	0	0	0	0	0
	Dilatation, collecting duct	0	1 (8.3)	0	0	0	0	0	0
	Protein casts	1 (8.3)	1 (8.3)	0	0	0	1 (8.3)	0	0
Liver	Microgranuloma	0	0	1 (8.3)	1 (8.3)	0	0	0	0
Lung with bronchus	Aggregation, foam cell	3 (25.0)	1 (8.3)	1 (8.3)	2 (16.7)	1 (8.3)	2 (16.7)	3 (25.0)	3 (25.0)
	Calcification, blood vessel	2 (16.7)	4 (33.3)	0	1 (8.3)	2 (16.7)	0	1 (8.3)	1 (8.3)
	Osseous metaplasia, intra‐alveolar	0	0	1 (8.3)	1 (8.3)	0	0	1 (8.3)	0
Mandibular gland	Ectopia, parotid gland	3 (25.0)	3 (25.0)	0	0	0	0	1 (8.3)	2 (16.7)
	Ectopia, sublingual gland	0	0	0	0	1 (8.3)	1 (8.3)	1 (8.3)	0
Medial iliac lymph node	Increased number of lymphocyte, sinus	–	–	–	–	–	–	1 (100.0)^a^	–
Ovary	Calcification	1 (8.3)	0	0	0	0	0	0	0
Pancreas	Cellular infiltration	1 (8.3)	0	0	0	0	1 (8.3)	0	0
Pituitary	Cyst, anterior lobe	1 (8.3)	0	0	0	0	0	0	0
	Rathke's pouch remnants	0	0	0	1 (8.3)	0	1 (8.3)	0	1 (8.3)
Skin/subcutis	Squamous cell papilloma	0	0	0	0	0	1 (8.3)	0	0
Spleen	Brown pigment deposit	7 (58.3)	4 (33.3)	0	0	5 (41.7)	3 (25.0)	5 (41.7)	6 (50.0)
	Extramedullary hematopoiesis	1 (8.3)	0	0	0	1 (8.3)	0	0	0
Sublingual gland	Ectopia, parotid gland	0	0	0	0	1 (8.3)	1 (8.3)	3 (25.0)	2 (16.7)
Thyroid	Ultimobranchial cyst	2 (16.7)	1 (8.3)	2 (16.7)	1 (8.3)	0	2 (16.7)	2 (16.7)	1 (8.3)

Values indicate incidence (%).

Findings are displayed for tissues with at least one incidence of abnormality from a total of 35 tissues examined in each animal.

The following tissues showed no abnormalities and were therefore not included in this table: bone, bone marrow, brain, cecum, colon, duodenum, esophagus, jejunum, mammary gland, mandibular lymph node, mesenteric lymph node, parathyroid, spinal cord, stomach, thymus, tongue, trachea, urinary bladder, uterus and vagina.

a Only one rat in experiment 2 was examined.

–Not examined.

Toxicologically relevant findings in MF‐exposed rats included astrocytoma in the brain, moderate congestion and severe necrosis in the caudate lobe in the liver, and squamous cell papilloma in the skin. In control rats, moderate cellular infiltrations in the pancreas and increased numbers of lymphocytes in the sinus of the medial iliac lymph nodes were found. However, these were isolated events, which did not occur uniformly within treatment groups.

## Discussion

We investigated the toxicity of IF MF exposure in rats because there are insufficient biological studies to allow accurate health risk assessment of such exposure, which occurs in the proximity of widely used commercial and domestic appliances. For 14 days or 13 weeks, groups of 12 male or female rats were exposed to either 20 kHz, 0.2 mT(rms) or 60 kHz, 0.1 mT(rms) sinusoidal MFs, or sham‐exposed, and a series of pathological examinations was performed in a treatment‐blind manner. Experiments were performed in duplicate in each frequency and exposure period.

In sham–sham experiments, except for some observations that showed no consistency in replicate experiments, the body and organ weights and hematological and clinical chemistry parameters were similar in the animals in the 20 kHz and 60 kHz MF‐facilities. This result supported the equivalency of the animal breeding environments in these facilities. Furthermore, the validity of this study was supported by the comparison that sham–sham and control data were similar and within historical data ranges reported for Crl:CD(SD) rats (Charles River Laboratories, 2001; Nakano *et al*., 2000). Most of the pathological findings in control rats in the subchronic MF exposure experiments are known to occur spontaneously in Crl:CD(SD) rats at the ages of those used in this study. Some histopathological findings, such as lymphoid cell aggregation in the prostate gland, appeared more frequently than would be expected from the literature. However, findings with toxicological relevance, such as swelling in the lymph nodes, occurred at a very low frequency.

In the MF exposure experiments, observed changes in male body weight during subchronic exposures were inconsistent in direction and not observed in females or in replicate experiments. Small differences at the start of the MF exposure period may have contributed to these group differences. No consistent significant changes in hematological or clinical chemistry parameters in MF‐exposed rats were associated with pathological findings identified at necropsy and/or subsequent microscopic examination. Pathological examinations identified changes in the MF‐exposed rats that could have occurred spontaneously, and toxicologically significant findings were sporadic. Although there were some interexperiment differences in the frequencies of these findings, these did not appear to be influenced by MF exposure, because they were balanced between groups within each experiment.

Among the histopathological findings in MF‐exposed rats, there were three toxicologically notable observations in animals subchronically exposed to a 60 kHz MF: brain astrocytoma and liver caudate lobe necrosis in male rats and muzzle nodules in a female rat. However, these animals did not exhibit any other neoplasm formation, necrosis or associated clinical sign. Astrocytoma is spontaneously observed in rats of this strain after the age of 46 weeks (Giknis *et al*., 2000; Ikezaki *et al*., 2011; Noto *et al*., 1999; Shibuya *et al*., 2001; Son and Gopinath, 2004; Tsunenari *et al*., 2000), but it is exceptional in young 19‐week‐old rats. The observed astrocytoma was limited to a single male rat, and did not occur in the replicate experiments, in females, or in rats exposed to 20 kHz MF. Based on this evidence, it is reasonable to conclude that the relatively early onset of this astrocytoma had little association with IF MF exposure. Because the liver necrosis appeared to be due to torsion and congestion of the lobe, and was limited to the caudate lobe, we considered this finding to have occurred by random chance. Squamous cell papilloma in the skin is also spontaneous in nature in aged rats of this strain (Baldrick, 2005; Giknis *et al*., 2000; Shibuya *et al*., 2001). Although the onset of this squamous cell papilloma was early in comparison to historic data sets, the incidence was isolated and was not replicated in other experiments. This evidence therefore indicates that it was unlikely to have been caused by IF MF exposure.

Recently, Ushiyama *et al*. (2014) reported that short‐term (1 h day^−1^) exposure of juvenile rats to a very intense (3.8 mT(rms), sinusoidal), 21 kHz MF for 14 days did not influence hematological, serological or immunological parameters. While the exposure time on each day was less than one‐twentieth of that used in the present study, the MF to which the animals were exposed was almost 20 times stronger and their results were in accordance with the hematological and clinical chemistry observations in our acute toxicity studies. Their study was designed to expose the entire rat body to an intense and uniform MF. Consequently, the coils and the designated exposure space were rather small relative to the rat body size. The rats were individually restrained in a plastic chamber, which limited the daily exposure time. The present study aimed to identify the possible health hazards associated with IF MFs and the target organs affected. Therefore, we also exposed the whole body of the rats to uniform MFs, but the animals were able to move freely in a cage while being exposed continuously for 22 h day^−1^ for up to 13 weeks, to maximize the opportunity for hazard identification.

The present study found that acute and subchronic exposure to 20 kHz, 0.2 mT(rms) or 60 kHz, 0.1 mT(rms) sinusoidal MFs showed no toxicity in rats. No MF frequency‐specific response was observed in any experiment, and these results do not support the hypothesis that IF MF exposure is toxic to mammals. As stated in the introduction, negative results were reported following acute and subchronic toxicity studies of 10 kHz MFs at up to 1.0 mT(rms) in mice (Robertson *et al*., 1996). Svedenstål and Johanson (1998a, 1998b), Kim *et al*. (2006) and Lee *et al*. (2006, 2010) also reported negative results from acute and chronic toxicity studies of 20 kHz MFs at up to 0.03 mT(pp) in rats and mice. Ushiyama *et al*. (2014) reported strong negative outcomes following short‐term exposure to 21 kHz MF at 3.8 mT(rms). Taken together with the results of the present study, mounting evidence indicates that IF MFs with frequencies below 60 kHz do not carry a significant health risk to mammals.

## Conflict of interest

The authors did not report any conflict of interest.
